# Prevalence of Sex-Related Chromosomal Abnormalities in a Large Cohort of Spanish Purebred Horses

**DOI:** 10.3390/ani13030539

**Published:** 2023-02-03

**Authors:** Sebastián Demyda-Peyrás, Nora Laseca, Gabriel Anaya, Barbara Kij-Mitka, Antonio Molina, Ayelén Karlau, Mercedes Valera

**Affiliations:** 1 Departamento de Producción Animal, Facultad de Ciencias Veterinarias, Universidad Nacional de La Plata, La Plata 1900, Argentina; 2 Consejo Nacional de Investigaciones Científicas y Técnicas (CONICET LA PLATA), La Plata 1900, Argentina; 3 Departamento de Genética, Universidad de Córdoba, 14071 Córdoba, Spain; 4 Department of Animal Reproduction, Anatomy and Genomics, University of Agriculture in Krakow, Mickiewicza 24/28, 30-059 Krakow, Poland; 5 Departamento Agronomía, Escuela Técnica Superior de Ingeniería Agromómica, Universidad de Sevilla, Ctra Utrera Km 1, 41013 Sevilla, Spain

**Keywords:** chromosomal abnormalities, horse, genomic, prevalence, SNP-array, cytogenetics

## Abstract

**Simple Summary:**

Horses are well known for the increased number of individuals carrying chromosomal abnormalities related to the sex pair, which have been identified as a major cause of idiopathic infertility. However, large-scale populational studies evaluating the occurrence of these chromosomal aberrations in commercial or wild populations are extremely scarce. We, therefore, performed a cytogenetic analysis on a large dataset of 25,237 individuals, gathered over a period of 24 months, using a two-step genomic-based diagnostic methodology. We first screened the entire population, analyzing the results of short tandem repeats parentage testing to determine individuals showing abnormal results. Thereafter, the positive samples, together with the individuals showing morphological abnormalities in the reproductive tract, were reanalyzed using a single nucleotide polymorphism (SNP)-based procedure to determine the occurrence of chromosomal abnormalities. Our results showed that the overall prevalence of individuals carrying chromosomal alterations was close to 0.05%, with blood chimerism and 64,XY sex-reversed mares the most common type of aberrations detected. In addition, one case of Turner and one of Klinefelter syndrome, as well as a small number of individuals carrying complex karyotypes, were also detected. However, these results should be taken with caution since the occurrence of X chromosome monosomy, a sex-related chromosomal aberration commonly reported in mares, cannot be screened using the methodology employed in this study. To our knowledge, this is the largest study performed aimed at determining the prevalence of the most important chromosomal abnormalities in the domestic horse.

**Abstract:**

Chromosomal abnormalities are largely associated with fertility impairments in the domestic horse. To date, over 600 cases of individuals carrying abnormal chromosome complements have been reported, making the domestic horse the species with the highest prevalence. However, studies analyzing the prevalence of chromosomal diseases in whole populations are scarce. We, therefore, employed a two-step molecular tool to screen and diagnose chromosomal abnormalities in a large population of 25,237 Pura Raza Español horses. Individuals were first screened using short tandem repeats parentage testing results and phenotypic evaluations. Those animals showing results suggesting chromosomal abnormalities were re-tested using a single nucleotide polymorphism (SNP)-based diagnostic methodology to accurately determine the chromosomal complements. Thirteen individuals showed a positive screening, all of which were diagnosed as chromosomally abnormal, including five 64,XY mares with sex development disorders (DSD) and four cases of blood chimerism (two male/female and two female/female cases). In addition, we detected one Turner and one Klinefelter syndrome and two individuals carrying complex karyotypes. The overall prevalence in the entire population was ~0.05%, with the prevalence of 64,XY DSD and blood chimerism ~0.02% and ~0.016%, respectively. However, the overall results should be taken with caution since the individuals carrying Turner syndrome (in full (63,X) or mosaic (mos 63,X/64,XX) forms) cannot be detected due to limitations in the methodology employed. Finally, the lack of agreement between populational studies performed using karyotyping or molecular methods is discussed. To our knowledge, this is the largest populational study performed evaluating the prevalence of the most common chromosomal abnormalities in the domestic horse.

## 1. Introduction

Chromosomal abnormalities related to the sex pair are a common genetic disease in domestic horses, *Equus caballus*. This knowledge is not new [1] but was demonstrated 30 years ago by Power [2], who compiled the karyotyping results of nearly 400 cases showing chromosomal aberrations. More recently, Bugno-Poniewierska and Raudsepp [3] established that chromosomal disorders are among the most common non-infectious causes of subfertility, infertility and congenital defects in the species, accounting for ~30% of horses with reproductive or developmental problems. However, cytogenetic analysis in such individuals is not a common practice and is far from being a systematic practice in large populations and/or commercial herds [4]. This lack of testing may make it difficult to determine the prevalence of chromosomal abnormalities in horses, especially since some individuals carrying chromosomal aberrations may be phenotypically normal, thus avoiding detection [5].

The most comprehensive and largest cytogenetic evaluation of a horse population existing to date was performed 15 years ago by Bugno, et al. [6], who reported a prevalence of chromosomal abnormalities related to the sex pair close to 2% by karyotyping 500 horses selected randomly. In contrast, Kakoi, et al. [7] conducted a large-scale analysis of 17,471 newborn light-breed foals in Japan using high-throughput molecular methods (instead of karyotyping) and found a much lower prevalence of chromosomal abnormalities, approximately 0.01%. Similarly, Anaya, et al. [8] used molecular methods to detect blood chimerism in 21,097 Pura Raza Español (PRE) horses, reporting a prevalence of 0.01%, which is 20 times lower than the previous existing reports. Given the discrepancy existing between results obtained using different methodologies, any additional data produced will help us to determine a more accurate rate of chromosomal abnormalities in the domestic horse.

Thirty years ago, Bowling, et al. [9] demonstrated the usefulness of short tandem repeats (STR) genotyping to detect chimerism in horses. More recently, this approach was further employed to detect the same type of chromosomal abnormality in an American Bashkir Curly [10] and several Pura Raza Español horses [8,11]. However, the standardized STR panel employed for parentage testing in the species includes only one ECAX marker and none located in the ECAY. Therefore, most of the aberrations associated with reproductive impairments, such as sex reversions (DSD) or ECAX monosomy [5], cannot be detected. To solve this issue, Kakoi, et al. [7] developed an extended STR panel with better coverage of sex chromosomes, detecting 17 individuals with abnormal complements in a large population of Japanese horses. However, more recently, our laboratory validated a novel, more accurate methodology for chromosome testing, based on the analysis of the information provided by single nucleotide polymorphism (SNP) genotyping arrays, in the PRE breed [12]. This method, which can detect almost any type of chromosomal abnormalities, was integrated as an auxiliary tool of the PRE breeding program in 2021.

The Pura Raza Español horse is one of the oldest and most important horse breeds bred in Europe [13], with more than 250,000 active individuals bred in over 60 countries in the present day [14]. Its studbook was created in 1912 and since then, it has been managed by the Real Asociación Nacional de Criadores de Caballos de Pura Raza Española (ANCCE), following a closed enrolment policy. For this reason, to be included in the PRE studbook, all individuals need to perform mandatory DNA testing to confirm the parentage assignation, as well as a phenotypic characterization to avoid the enrolment of individuals with morphological variations forbidden by the PRE breeding program bylaws. Since 2021, all PRE individuals showing any reproductive abnormality in the mandatory pre-enrolment phenotypic assessment or those whose STR-based parentage test results showed any abnormal or incongruent results (more than 2 alleles per loci or incompatibility between genotypes and phenotypic sex) are being flagged as presumptive carriers of chromosomal abnormalities and submitted for further investigation using SNP genotyping. Two years later, almost 25,237 horses have now been screened in one of the largest cytogenetic studies conducted on the species.

Here, we present the results of the screening for sex-related chromosomal alterations in a large population of PRE horses, with the aim of establishing a more accurate estimation of the prevalence of chromosomal abnormalities in the species.

## 2. Materials and Methods

### 2.1. Animal Samples

All the individuals analyzed in this study are included in the screening program for chromosomal abnormalities of the Real Asociación Nacional de Criadores de Caballo Pura Raza Español (ANCCE) studbook. All of them provided blood samples collected by the ANCCE official veterinary services, according to the breeding association standard protocol for parentage testing, before their enrollment in the studbook. During the last 24 months, 25,237 individuals were evaluated (12,569 in 2021 and 12,668 in 2022).

### 2.2. Genotyping and Chromosomal Analysis

DNA was obtained from biological samples (either whole blood or hair bulbs) using regulation extraction kits from Qiagen (Madrid, Spain). Thereafter, the samples were first genotyped using the 17 STR panel for determining parentage in horses proposed by the International Society for Animal Genetics (ISAG) in a multiplexed determination using a set of commercially-available fluorescent-labeled primers (StockMarks kit for horses, PE Applied Biosystems, Foster City, CA, USA). In the same reaction, we determined the presence or absence of ECAX and ECAY gene-specific *amelogenin* markers using a slight modification of the PCR reaction proposed by Hasegawa, et al. [15]. Finally, the genotyping and allele calling was performed by capillary electrophoresis using an Applied Biosystems 3130xl DNA sequencer (ANCCE, Spain).

Any individuals who showed abnormal genotyping results (more than 3 alleles in different loci or those showing a discrepancy between the phenotypic and genotypic sex in the ECAX specific marker (LEX003) or AMELX and AMELY fragments, according to Anaya-Calvo [16]) or which showed phenotypic abnormalities in the external reproductive organs were further genotyped using a medium density SNP array chip (Equine GGP 70K, Neogen Inc, Scotland, UK). Finally, the chromosomal complements of these animals were determined according to the methodology validated in the PRE by Pirosanto, et al. [12]. In addition, these individuals were reinspected phenotypically by an official ANCCE veterinarian in situ to confirm the phenotypic sex and to determine the presence or absence of phenotypic abnormalities in the reproductive tract.

## 3. Results

Thirteen individuals (0.051% of the total population analyzed) were submitted for chromosomal analysis during the 24-month period (Table 1). Among them, three were submitted due to the existence of morphological abnormalities in the external gonads, four showed three or more alleles in a single locus in the parentage testing STR panel and six showed incongruences between phenotypic and genotypic sex. In all the cases, the presence of chromosomal abnormalities was confirmed by the SNP genotyping (Table 1).

**Figure 1:**
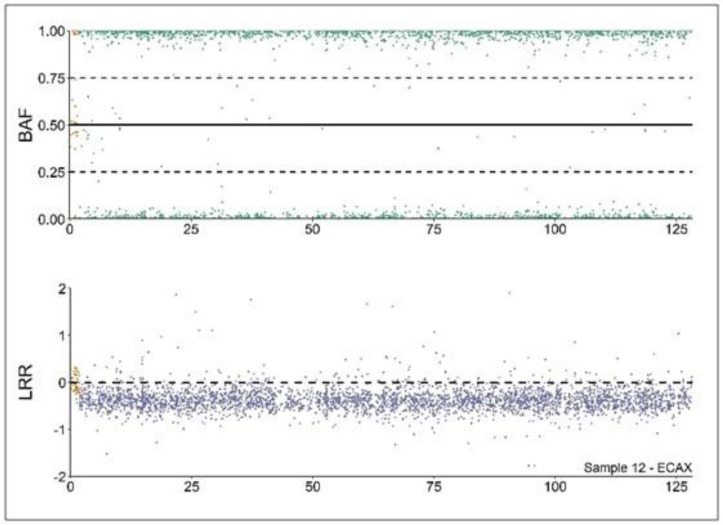
**Copy number alterations (CNA) analysis profile of a 64,XY DSD mare**. Analysis of the B allele frequency (BAF) and Log R ratio (LRR) values from a 64,XY DSD individual according to [10]. BAF values (upper part) depicting hemizygous markers (close to 0 or 1) in the non-pseudoautosomal region (XPAR, green). Conversely, the XPAR (in yellow) is mostly heterozygous (values close to 0.5). LRR values (lower part) are close to 0 in XPAR (in yellow), depicting diploidy. On the contrary, values in the non-XPAR region (in purple) are close to −0.5, depicting monosomy.

**Figure 2:**
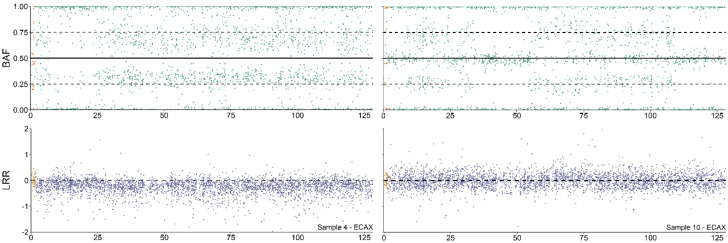
**Copy number alterations (CNA) analysis profile of 64,XY/64,XX and 64,XX/64,XX blood chimeras.** Analysis of the B allele frequency (BAF) and Log R ratio (LRR) values of the ECAX from 64,XY/64,XX (Sample 4, left part) and 64,XX/64,XX individuals (Sample 10, shown on the right of the figure) according to Pirosanto, et al. [12].

The results show that 64,XY DSD sex reversal mare (see Figure 1) was the most common syndrome detected (five cases, 0.02%), followed by blood chimerism (four cases, 0.015%). Interestingly, two of them were male/female chimeras, whereas the remaining two were female/female chimeras (see Figure 2).

In addition, we detected a Klinefelter horse (65,XXY), a Turner’s mare (63,X) and two mosaicisms (63,X/64,XX and 63,X/64,XY). Finally, the overall prevalence of individuals carrying chromosomal abnormalities in the PRE during the last 24 months was 0.051% (13/25,237). However, it should be mentioned that individuals carrying 63,X ECAX monosomy or low-level chimerisms could be screened using the diagnostic pipeline employed in this study and therefore, the overall prevalence is most likely underestimated.

## 4. Discussion

It is well established that the domestic horse is the domestic species with the highest number of individuals carrying chromosomal alterations [17], most of which are associated with reproductive failures and abnormal phenotypes [5]. This knowledge is mostly built on the compelling number of cases reported to date, such as the ~400 horses compiled by Power [2] 30 years ago or the 214 horses analyzed by the TAMU cytogenetic lab over the last 20 years [3]. However, large-scale cytogenetic surveys analyzing whole populations are scarce. Here, we establish the incidence of the most important chromosomal abnormalities in a population of 25,237 Pura Raza Español horses.

Several reasons have been suggested for the lack of large cytogenetic surveys in horses, such as the inability of field practitioners to establish an association between infertility and chromosomal failures, but also the scarce availability of commercial laboratories providing karyotyping services in the species [4]. In addition, the classical cytogenetics techniques are slow, expensive and require the collection and shipping of biological samples for cell culture. However, 20 years ago, Kakoi, et al. [7] proposed the viability of the use of STR parentage genotyping as a screening tool to detect chromosomal abnormalities in horses. Based on this idea, we established a relationship with the Pura Raza Español breeding association, which allowed us to detect several individuals carrying chromosomal abnormalities in the PRE breed, including chimerisms [11], DSD sex reversal horses [18] and complex karyotypes [19], such as normal and abnormal cell lines originating in two different individuals in the same horse. However, it was not until more recently, with the validation of an SNP-based methodology [12], that chromosomal screening was integrated into the breeding program. Since then, more than 25,000 horses have been screened, thus producing one of the most comprehensive datasets analyzed to date.

So far, two large-scale studies have been performed to analyze horse populations from a cytogenetic point of view. In 2005, Kakoi, et al. [7] analyzed 17,471 light-breed Japanese horses using a molecular approach based on STR parentage genotyping, similar to the one used in our study. The authors were able to detect 18 individuals with presumptive chromosomal abnormalities in the sex pair, establishing a prevalence of close to 1/1000. In the second step, they employed an extended ECAX/ECAY STR panel, thus confirming the existence of 13 Turner mares (63,X, 0.07%), 4 Klinefelter horses (65,XXY, 0.02%) and a 65,XXX mare (0.006%). However, only four of these cases could be confirmed by karyotyping due to the lack of availability of biological samples. Two years later, Bugno, et al. [6] karyotyped 500 young Polish horses from several breeds, reporting 10 individuals (2%) with chromosomal abnormalities. Among them, the authors reported a male/female chimera (64,XX/64,XY, 0.2%), one Turner syndrome (63,X, 0.2%), 7 ECAX mosaicisms (63,X/64,XX, 1.4%) and an individual with an autosomal trisomy in the ECA31. Our study agrees with the results provided by Kakoi, et al. [7], probably due to the fact that they were performed using a similar diagnostic methodology in which a large population was first screened and then only the horses suspected of carrying chromosomal abnormalities were re-analyzed for confirmation. In contrast, the later study karyotyped all 500 horses, with the most common abnormality reported being 63,X/64,XX mosaicism, which was barely detected in our study (just one case). Since this later syndrome is difficult to screen using molecular methods [20], it may explain the differences observed between these different approaches. However, it is also worth mentioning that an overall incidence equal to that reported by [6] would be equivalent to detecting nearly 500 horses carrying chromosomal abnormalities in our PRE dataset, which is far higher than the figure we found. In this context, Shah, et al. [21] reported a 33% discordance rate between karyotyping and molecular methods in the analysis of human miscarriages, with the appearance of chromosomal artifacts due to prolonged culture being one of the potential causes. This lack of consensus was even more noticeable when the growth of cultured cells was stimulated with phytohaemagglutinin (PHA), which resulted in an increased number of abnormalities in comparison with the same samples analyzed from unstimulated cultures [22]. However, we previously compared the results of karyotyping and molecular methods in 30 PRE horses (10 carrying chromosomal abnormalities) with 100% of accuracy [23]. For this reason, we hypothesize that this could be an additional cause to explain the differences observed among studies, such as the breed effect, or maybe another, which was not analyzed in the present report.

One of the key questions about the use of molecular methods for detecting chromosomal abnormalities is their accuracy in determining the presence of mosaicisms/chimerisms in which the percentage of abnormal cells is low [24]. This is particularly important since Power [2] reported that 15% of the 401 individuals carrying chromosomal abnormalities were 63,X/64,XX mares. These limitations were recently demonstrated in horses by Szczerbal, et al. [20] using a highly accurate technique (ddPCR) and by our group using an array-based SNP methodology [12]. However, this is also a well-known problem in humans, where the ability to detect low levels of mosaicism with less than 10% of abnormal cells is questionable [25]. Pienkowska-Schelling, et al. [26] recently reported a substantial increase in the rate of ECAX mosaicism (63,X/64,XX) in fertile mares by performing classical karyotyping. This finding agrees (although the incidence was different) with the reports demonstrating that some of these individuals can develop normally and produce offspring [6,27]. Nevertheless, most of the cases of 63,X/64,XX described are associated with subfertility [17]. Despite the fact that the use of molecular methods for this particular syndrome is still in its infancy, its importance may be limited in the whole population in comparison with the other chromosomal abnormalities reported to date [2,3].

One important limitation of this study worth mentioning is the inability to screen Turner’s syndrome (63,X; ECAX monosomy) using parentage STR markers since the standardized test performed worldwide includes only one STR located in the ECAX. In this context, Bugno, et al. [6] reported a prevalence value of 0.2% (1 in 500) for this abnormality in the whole population (1/500). Similarly, several reports [2,17,28,29] have established that ECAX monosomy can account for approximately 30/40% of all the chromosomal abnormalities detected in horses. In contrast, Kakoi, et al. [7] reported a prevalence of this abnormality close to 0.075% (13/17,471), although they were only able to determine those individuals in which the foal’s ECAX was of paternal origin. Since we are not able to estimate the incidence of such aberrations in PRE, we extrapolated the results obtained in other breeds (30% of incidence among chromosomal abnormalities) to our dataset to obtain a hypothetical incidence for comparison purposes. This estimated value (0.016%) is ten times lower than that reported by Bugno, et al. [6] but more in line with that reported by Kakoi, et al. [7] as well as with the incidence of this syndrome in humans (0.04%, according to Bondy [30]), although a large-scale study would be needed to determine the real prevalence of this syndrome in horse populations. Here, SNP-based methodologies can accurately detect mares carrying 63,X complements [12]. Given the introduction of SNP arrays and the genotyping of horses in breeding programs [31,32,33]), we should expect to find an increase in the detection of this chromosomal abnormality in large populations of horses within the next few years.

Disorders in sex development constitute another common syndrome in horses [5]. Among these, the most common is the sex-reversal mare (64,XY DSD), in which the individual shows a mare phenotype instead of carrying male chromosomal complements [34], even in the PRE breed [18]. In our study, we used a combination of an ECAX STR marker (LEX003) together with determining two fragments of the sex-related *amelogenin* gene (AME), which reduces the possibility of misdiagnosis close to null. However, Martinez, et al. [35] reported a very low percentage of inconsistencies in the AMEY testing (8/100,000) in a large population of horses, suggesting that they might be the result of a translocation (in the paternal line) from the Y to another chromosome. In our case, all the individuals showing a positive AMEY amplification were confirmed as 64,XY DSD by SNP genotyping (6/6). In terms of prevalence, Power [2] stated that 28% of the individuals carrying chromosomal abnormalities showed a 64,XY karyotype, whereas Bowling, et al. [28] detected 22 individuals showing this chromosomal arrangement among 98 mares. In our case, the incidence of 64,XY DSD among individuals showing abnormal karyotypes was ~40%. However, 63,X was not included in this study and therefore, results may, to some extent, be overestimated. Interestingly, we determined a prevalence of 0.02% 64,XY DSD in the whole population analyzed. This result is in full agreement with the largest screening programs performed for this syndrome in the species, which reported the same value [35]. Interestingly, neither [6] nor [7] reported any individual showing a DSD in their populational studies. However, the fact is that analysis of almost 235,000 individuals, together with the long history of cases reported during the last 40 years, suggest that 64,XY DSD is one of the major chromosomal abnormalities in the domestic horse.

Finally, we were able to detect four cases of blood chimerism in the whole population (4/25,237; 0.016%). These results agree with Anaya, et al. [8] (0.024%), who analyzed ~21,000 PRE foalings but were ten times lower than those reported by Bugno, et al. [6]. Since the electropherogram pattern obtained in STR parentage can only be caused either by blood chimerism or by cross-contamination of the sample, the use of an additional DNA sample is mandatory. In our case, the results obtained using DNA obtained from hair bulbs were normal (not chimeric). However, SNP genotyping also allowed us to detect 64,XX/64,XX blood chimeras, which cannot be detected using classical or molecular karyotyping. Since it is perfectly possible to misdiagnose chimeric samples as normal, we believe that the occurrence of one case of blood chimerism every 5000 foals is a reasonable and reliable estimation for the domestic horse.

## 5. Conclusions

Chromosomal abnormalities associated with the sex pair are a noticeable problem in horse fertility. In this study, we demonstrated that the use of a combined technique, including STR and SNP genotyping, can detect most of these genetic abnormalities in horses at an early age. We detected the existence of chromosomal abnormalities in 0.05% of the 25,237 PRE individuals analyzed over the 24-month period within the official breeding program. However, we were also able to estimate a reliable prevalence for specific chromosomal abnormalities, such as 64,XY DSD and blood chimerism, by analyzing one of the largest datasets to date. However, the overall prevalence could be underestimated because of our inability to screen 63,X individuals due to methodological limitations. Finally, we suggest that the increasing use of SNP genotyping within breeding programs will allow us to detect most of the individuals carrying chromosomal abnormalities in the next few years in a reliable, systematic way.

## Figures and Tables

**Table 1:** Individuals carrying chromosomal abnormalities during the 24-month period in the PRE breed.

Individual	Year	Phenotypic Sex	Parentage STR	SNP Genotypìng
1	2021	Intersex	Female	63,X
2	2021	Male	Multiallellic	chi 64,XX,64,XY
3	2021	Female	Multiallellic	chi 64,XX,64,XX
4	2021	Female	Multiallellic	chi 64,XX,64,XY
5	2021	Female	Sex incongruence	64,XY
6	2021	Female	Sex incongruence	64,XY
7	2021	Intersex	Normal female	mos 63,X/64,XX
8	2022	Female	Sex incongruence	64,XY
9	2022	Male	Sex incongruence	65,XXY
10	2022	Female	Multiallellic	chi 64,XX,64,XX
11	2022	Intersex	Male	mos 63,X/64,XY
12	2022	Female	Sex incongruence	64,XY
13	2022	Female	Sex incongruence	64,XY

A short tandem repeat (STR) based parentage test was performed according to Demyda-Peyras, et al. [11]. Single nucleotide polymorphism (SNP) genotyping analyses were performed according to Pirosanto, et al. [12]. N.d.: not detected.

## Data Availability

All the data analyzed in this study belong to the Pura Raza Española breeding program. The ANCCE allows its use for scientific purposes under a specific agreement of collaboration with the MERAGEM group. Therefore, the genetic and genomic data employed in this study is not publicly available. Access can be granted by request to the corresponding authors upon agreement with the ANCCE.
